# Giant unilamellar vesicles as a model system for studying ion transport

**DOI:** 10.1007/s12551-025-01342-6

**Published:** 2025-08-09

**Authors:** Marcus Fletcher, Yuval Elani, Ulrich F. Keyser, Ran Tivony

**Affiliations:** 1 Department of Chemical Engineering, Imperial College London, Exhibition Road, London, SW7 2AZ UK; 2 Cavendish Laboratory, University of Cambridge, J.J. Thomson Avenue, Cambridge, CB3 0HE UK; 3 Department of Chemical Engineering, Ben-Gurion University of the Negev, Beer-Sheva, 84105 Israel

**Keywords:** Ion transport, Artificial cell membrane, Giant unilamellar vesicles

## Abstract

Cellular activity depends on constant flux of ions across biological membranes. Artificial membrane models like planar lipid bilayers and liposomes are ideal for studying membrane transport phenomena as they are free of the structural complexity of cells and allow examination of transport processes under tightly controlled conditions. Over the last decades, artificial membrane-based techniques like single-channel recording and fluorescent monitoring of transport through bulk lipid vesicle suspensions have revealed many molecular mechanisms of transport. Recently, giant unilamellar vesicles (GUVs), cell-sized liposomes, have emerged as an important tool for studying cellular processes, including ion transport. The principal advantage of GUVs derives from their micron scale, which enables ease of visualisation and manipulation using microscopy and microhandling. For that reason, GUVs have also become the state-of-the-art for recapitulating a host of cell structures and functions for the purpose of developing artificial cells. Taken together, GUVs represent a promising biomimetic system to elucidate ion transport mechanisms and unravel the association between ion fluxes and various cellular processes such as neuronal transduction, nutrient uptake, electrochemical gradient development. Nevertheless, despite their great potential as a model system, the use of GUVs in ion transport studies is still limited. The aim of this review is to outline recent GUV-based ion transport studies, describe the current techniques for measuring ion transport in GUVs, compare the utility of GUVs relative to other available techniques such as single-channel current recording, and explore the potential of using GUVs to investigate complex ion transport processes.

## Introduction

All living cells strive to maintain ionic gradients across their membrane by actively regulating transmembrane fluxes of ions (Ames 2000; Morth et al. 2007). This process of electrochemical gradient formation provides cells the required energy for driving vital cell processes like ATP regeneration, nutrient uptake, and membrane potential maintenance (Dubyak 2004; Kulbacka, et al. 2017). The production of energy through charge separation across biological membranes has inspired researchers to ask how to design synthetic cell systems with similar capabilities. Such systems will enable the design of autonomous and stimuli responsive therapeutics (Peng et al. 2024), protein factories (Krinsky et al. 2018) and synthetic cells that can influence the electrical state of living cells—a variable whose relevance to tissue development is becoming increasingly clear (Levin 2017). Recently, Berhanu et al. demonstrated the coupling of transient ATP regeneration to protein synthesis inside artificial cells powered by photo-induced pH gradients (Berhanu et al. 2019). However, ATP generation efficiencies in their system remained low and short-lived when compared to cells which can sustain out-of-equilibrium ATP regeneration cycles. Indeed, whilst artificial cells are being engineered with increasingly cell-like functions, their ion transport capabilities remain rudimentary. The disparity between cellular and artificial cell ion transport capabilities warrants an evaluation of our current understanding of ion transport mechanisms.

**Fig. 1:**
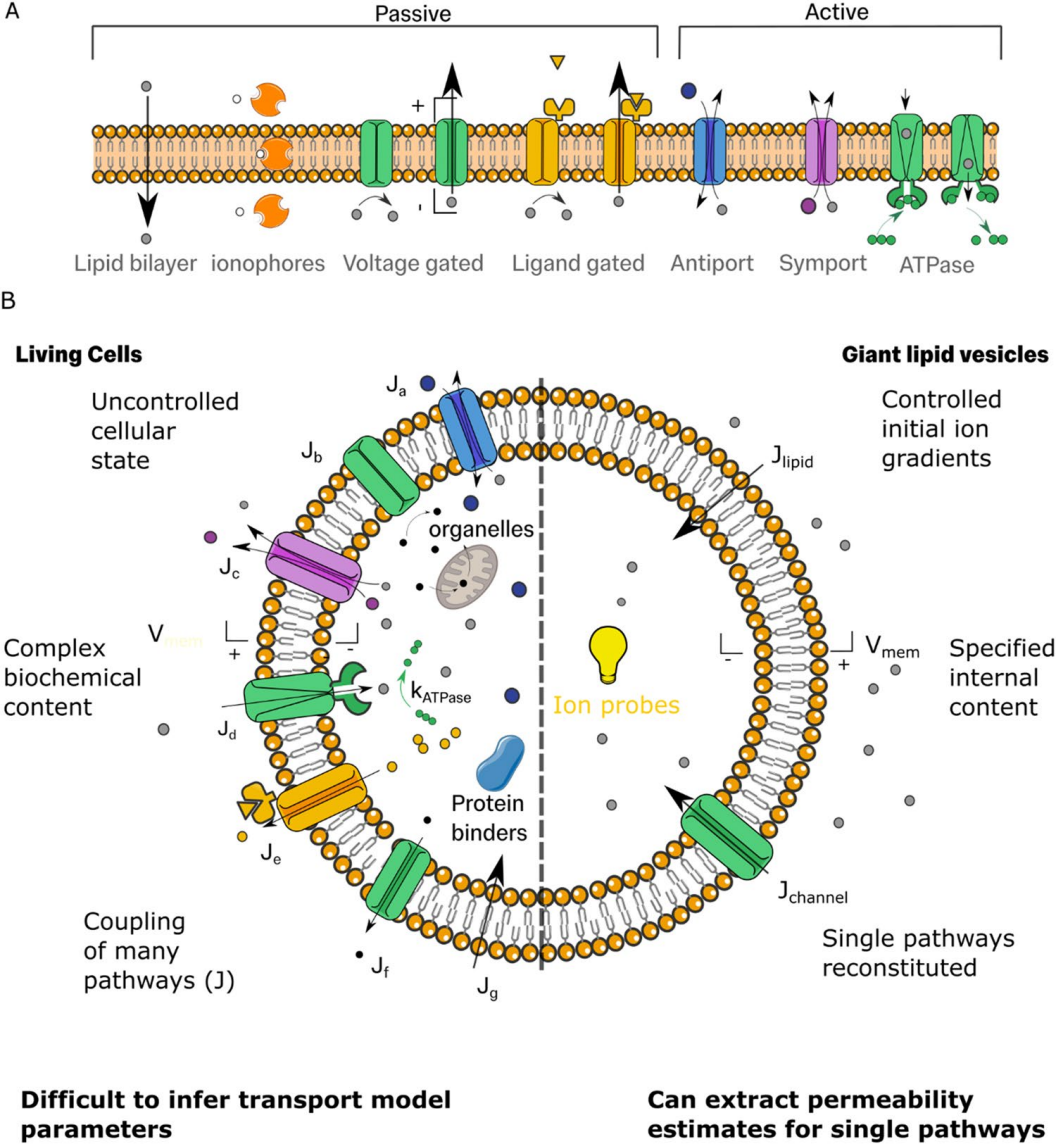
Ion transport pathways in living cells and artificial cell mimics.** A** Schematic illustrating different types of transport pathways by which ions can cross the membrane. **B** Ion transport pathways in living cells (left) and in artificial cell mimics (right). The measured net flux density, $J$, of an ion is the sum of the fluxes of each individual pathway ${J}_{i}$ across the membrane. To model ion transport pathways, we aim to measure the dependence of ${J}_{i}$ on ionic concentration gradients and the membrane voltage, ${V}_{\text{mem}}.$ In artificial cells, individual pathways can be reconstituted and studied under controlled membrane and solution compositions. However, in living cells, ion transport measurements only produce the net result of many coupled pathways both across the lipid bilayer and transport proteins. Cartoons of lipids adapted from lipid-yellowgray icon by Servier https://smart.servier.com/ licensed without endorsement under CC-BY 3.0 Unported https://creativecommons.org/licenses/by/3.0/

Decades of investigation into ion transport have revealed a complex picture of cellular ion transport where ions can pass through several distinct pathways (Dubyak 2004). Several of these are illustrated schematically in Fig. 1A. Ions can transport passively down their electrochemical gradients both through the lipid bilayer and within aqueous filled protein pores (Kulbacka et al. 2017). Some of these ion pores can be opened and closed through electrical and chemical gating mechanisms to cause nonlinear responses in protein pore permeabilities (Armstrong and Hille 1998). At the same time, cells can actively transport ions against their gradients using ATP-driven ion pumps (Morth et al. 2007).

In cells, ion transport involves the tight coupling of several pathways (Fig. 1B, left). As a result, understanding how cellular activity emerges from multiple coupled ion pathways requires the study of the contribution of each pathway to the overall behaviour. However, examination of specific transport pathways in live cell studies is highly challenging as pathways cannot be studied in isolation. Moreover, elucidating the molecular mechanism of membrane transport pathways in cells is inherently complex, as systematic variation of components like ion channels, lipids, and ligands is often constrained by the strict regulation of cellular homeostasis. This lack of precise mechanistic insight directly restricts our ability to elucidate ion transport phenomena and engineer artificial cell systems with cellular ion transport capabilities.

An alternative strategy for studying ion transport is to build artificial membrane mimics (Fig. 1B, right) which incorporate individual ion transport pathways (Burns et al. 2013; Tivony et al. 2022; Ruppelt et al. 2024; Fletcher et al. 2022). With these artificial systems, fundamental theoretical assumptions can be scrutinised under controlled conditions and model parameters of individual pathways, like ionic permeabilities, can be accurately determined. Furthermore, several pathways can be progressively combined one by one to investigate complex transport processes and their association with specific cellular activities.

Several different broad classes of artificial membrane studies have been applied to study ion transport: i) single-ion channel recordings (SCR), where electrical contact is made across two aqueous compartments bounded by a planar membrane, or membrane patch (Sakmann and Neher 1995) and ii) monitoring of transport across nanometric liposomes (with typical diameters ranging from 50 to 500 nm) using fluorescent ion indicators or ionic radioisotopes (Nimigean 2006). In recent years, GUVs, micron-sized liposomes, have emerged as an advanced and complementary membrane system that opens new avenues for investigating and quantifying ion transport and associated parameters (Valkenier et al. 2015; Garten et al. 2017; Tivony et al. 2022; Fletcher et al. 2022).

It is the aim of this review to outline insights gained from GUV-based ion transport studies and compare their current advantages and limitations in comparison to traditional techniques. Briefly, we will compare GUVs to traditional membrane models for ion transport and discuss their potential as a model system. Then, we will describe recent technical progress in GUV-based ion transport measurements, as well as in recapitulating different transport pathways. Finally, we will discuss the path towards reconstituting complex cell-like ion transport systems in giant vesicles.

## Comparison of GUVs with other artificial membrane transport systems

Table 1 summarises the main advantages and disadvantages of prominent artificial membrane systems that are used to study ion transport across biological membranes. SCR measurements using planar bilayers remain the state of the art for scrutinising relationships between molecular structure of ion channels and their transport properties, as single-channel conductances and selectivities can be precisely quantified (Sakmann and Neher 1995). Moreover, key transport variables such as membrane potential, *V*, and ionic concentrations in each aqueous compartment can be precisely controlled. However, low throughput, short membrane lifetimes, high technical overheads, and unphysiological membrane mechanics have meant LUV studies have been widely adopted in ion transport studies as well.

**Table 1:** Comparison of GUVs with other common artificial membrane models for ion transport studies

Model system	Transport measurement	Advantages	Disadvantages
GUVs	Fluorescence, current recording	Medium to high throughput.	Transport is measured across multiple membrane channels.
		GUV preparation can be performed using a wide range of techniques with varying levels of complexity.	Insertion of membrane proteins (e.g., ion channels or pumps) can be technically challenging.
		Single-vesicle measurement.	Not suitable for capturing short-lived events like gating.
		Suitable for short- and long-term measurements (minutes to days).	
		Defined lamellarity, volume, and surface area.	
		Transport measurements can be coupled with other biological activities and membrane transport processes.	
		Accurate determination of ion concentration gradients, permeability rates, electrochemical gradients, and selectivity.	
SUVs/LUVs	Fluorescence, radioactivity	High throughput.	Vesicles are heterogeneous in lamellarity, volume, surface area, and lipid content.
		Vesicle preparation is straightforward.	Nanometric size of vesicles might introduce curvature effect.
		Suitable for short- and long-term measurements (minutes to days).	Not suitable for capturing short-lived events like gating.
		Bulk transport measurements require minimal resources and are straightforward to implement.	In bulk experiments, ion concentration gradients cannot be determined accurately.
		Suitable for long period measurements of slow transport events.	
		Single vesicle measurement is possible.	
Planar lipid bilayers	Current recording	Single channel recordings.	BLMs are pressure sensitive and may break down due to applied voltages ( Gutsmann et al. 2014 ).
		No curvature effect.	Not suitable for long-term (hours-day) transport measurements.
		High temporal resolution (µs to ms).	Low throughput.
		Suitable for capturing short-lived gating events.	Technically demanding.
		Accurate quantification of ion permeability rates and selectivity.	

Note also that a third, related model system is the purple membrane from *Halobacterium salinarum* ( Govindjee et al. 1980 ). Unlike the aforementioned techniques, the purple membrane is purified from living cells. However, under specific growth conditions, purple membrane patches can be grown which contain a single protein (light stimulated ion pumps called proteorhodopsins) at high density (Krebs 2000). Consequently, these membrane patches can be included in synthetic vesicles to study the action of active ion transport in controlled conditions. Given the purple membrane’s specific application to proteorhodopsin studies, we do not consider it further in this review but recommend the following references to interested readers ( Stoeckenius et al. 1979; Krebs 2000).

LUVs are simple to form and are stable at the timescale of days. LUVs can be loaded with fluorescent ion indicators, or with radioisotopes of pertinent ions, and transport measured by monitoring the total solution fluorescence, or radioactivity (Nimigean 2006). LUV-based studies are popular as they can be performed with standard lab equipment (Deamer 1987). Consequently, LUV-based ion transport studies have been useful for establishing basic passive transport mechanisms across lipid bilayers made from a wide range of lipids (Paula et al. 1998). However, the nanoscopic size of LUVs limits the accurate quantification of crucial parameters, like ionic permeabilities, as it is difficult to precisely determine ionic concentrations inside and outside LUVs locally. Moreover, LUV techniques provide the bulk average over populations of liposomes, which are often very heterogeneous in size, lamellarity, and lipid content (Tivony et al. 2022). LUV studies are also limited when studying ion channel-associated transport in comparison to SCR due to the fact that LUV ion transport quantification is typically dependent on the number of ion channels in the LUV, which is difficult to accurately quantify (Miller 1984; Deamer 1987). Consequently, permeabilities per ion channel elucidated from LUV studies may have significant errors.

Giant unilamellar vesicles (GUVs) may offer a useful alternative artificial cell system for studying transport by combining useful properties from both planar bilayers and LUVs into one system. For example, similar to planar bilayers, the aqueous compositions on either side of the membrane can be precisely controlled and measured (in the case of GUVs, aqueous compositions can be visualised using fluorescence microscopy (Valkenier et al. 2015)). In addition, like LUVs, GUVs can be made in large sample sizes (1000s). In contrast to LUVs, however, microhandling techniques like microfluidics and microaspiration can be used to manipulate GUVs at a single vesicle level, enabling the collection of rich datasets that reflect the distribution of transport parameters across individual vesicles (Schaich et al. 2019; Cama et al. 2015). Moreover, GUV compositions can be controllably varied (Fletcher and Elani 2025), offering the opportunity to efficiently generate libraries of GUV compositions and study the effects of compositional features on ion transport.

An area where GUVs have immense potential to excel is in studying the coupling between ion transport and other cellular processes. Increasingly, sophisticated artificial cell models are being established based on GUVs, which can replicate complex cellular functions synthetically, e.g., express proteins, divide, and replicate biochemical pathways (Elani et al. 2015; Lira 2025). Consequently, there is significant potential to leverage the expanding library of artificial cell models to study the interactions of ion transport with other vital cell processes. Yet, GUV ion transport studies are still in their nascency and currently possess several limitations in comparison to traditional SCR. In the following sections, we will introduce the recent experimental strategies employed to study ion transport on GUVs.

## Experimental strategies to monitor ion transport across GUVs

Measuring ion transport across GUV membranes may be broadly divided into two approaches: i) coupling electrical currents to ionic transport fluxes and ii) monitoring concentrations of specific ions inside and outside the vesicle.

Monitoring electrical current across GUVs has been achieved by adapting the ‘patch clamp’ technique, which has been used for several decades to study membrane transport in cells (Garten et al. 2015; Cama et al. 2015). In this method, a micropipette with a pore tip of < 10 $\mu$ m is used to aspirate a GUV by coupling a negative pressure source to the other end of the pipette. At the same time, electrodes positioned both within the pipette and in the external solution allow the generation of voltage across the GUV membrane. When a high resistance seal is made between pipette and membrane, the overall circuit resistance can be dominated by the large resistance of membrane systems embedded with small nanosized ion channels. In this configuration, researchers have used electrical current measurements to estimate the density of open ion channels (Cama et al. 2015). Additionally, by carefully tuning the number of ion channels in the GUV membrane, previous studies have demonstrated that single-channel current measurements are possible (Goulian et al. 1998). This means that, in principle, quantities typically measured using SCR in planar membranes could be similarly measured in GUVs, such as pore opening probabilities and lifetimes. However, stability of aspirated GUVs, specific requirements for channel open-state lifetimes, and an inability to directly control the voltage either side of the GUV membrane limit the accurate quantification of ion channel properties such as channel conductance (Goulian et al. 1998).

**Fig. 2:**
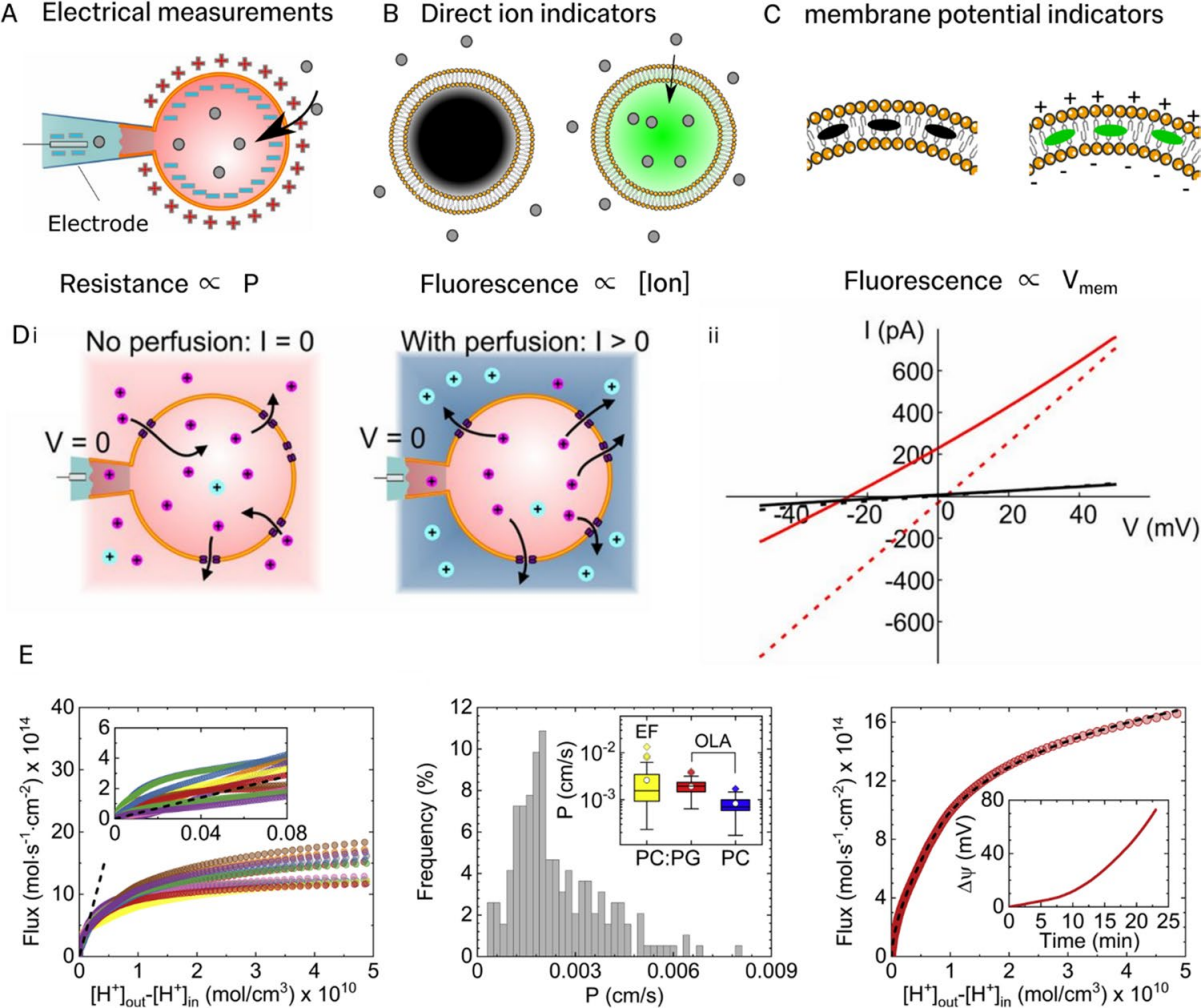
Recent advances in quantifying ion transport in artificial cell models**. A** Transport rates of ions across giant unilamellar vesicles (GUVs) have been measured using electrical methods inspired by the cell-based patch clamp technique. A GUV is aspirated using a micropipette to form a high (electrical) resistance seal. Ionic transport properties of membrane systems can be extracted from I-V relationships for individual GUVs. **B** Fluorophores whose fluorescent properties are dependent on ionic concentrations of a specific ion can be used to measure ion transport rates by monitoring GUV fluorescence intensity. **C** Fluorescent constructs whose fluorescence intensity is dependent on membrane potential. **Di** Garten et al. measured I-V curves across GUV membranes before (left) and after (right) inducing salt asymmetry using perfusion (Inside GUV: 95mM Na^+^, 5mM Chol^+^, perfusion solution: 5mM Na^+^, 95mM Chol.^+^). Note, however, there is significant uncertainty in external solution concentration as the perfusion does not completely displace the external solution. **Ii** Using this method, the authors additionally measured reversal potentials (right) across GUVs with (red, solid) and without (black, solid) gramicidin A after exchange of potassium in the external solution for sodium. With symmetric ion conditions gramicidin A GUVs have a zero reversal potential (red, dotted). Reproduced with permission from (Garten et al. 2017) (Copyright (2016) National Academy of Sciences). **E** Tivony et al. determined the passive proton flux across lipid bilayers vs. concentration difference characteristic from monitoring proton concentrations both within and outside single GUVs (left). The flux vs. proton concentration difference characteristic is nonlinear due to the build up of membrane potential, Δ*Ψ*, associated with different transport rates of protons and chloride, following the addition of hydrochloric acid (HCl). The authors identified linear regions in the flux vs. proton concentration gradient profiles for the early period of transport (left plot), corresponding to periods of low membrane potential. From these linear regions the authors estimated the proton permeability coefficient, ${P}_{{H}^{+}}$, for populations of GUVs. Using Eq. (4), the authors compared permeability values for lipid vesicles formed by distinct methods: electroformation (EF) and octanol-assisted liposome assembly (OLA), for two lipid compositions, DOPC:DOPG 3:1 and DOPC (middle plot). Using mean population values for ${P}_{{H}^{+}}$, the membrane potential, ΔΨ, variation during transport (right plot, inset) could be evaluated from relationships in flux vs concentration gradient plots, using Eq. (1). Taken with permission from reference (Tivony et al. 2022). Cartoons of lipids adapted from lipid-yellowgray icon by Servier https://smart.servier.com/ licensed without endorsement under CC-BY 3.0 Unported https://creativecommons.org/licenses/by/3.0/

A recent development was the introduction of whole-GUV patch clamping, where a voltage pulse is applied to aspirated GUVs to perforate the membrane and allow direct electrical contact to the GUV lumen, as illustrated in Fig. 2A (Garten et al. 2017). In their study, Garten et al. showed that coating glass micropipette pores with the protein casein stabilised the GUV in this configuration, where otherwise the lipid membrane would rapidly spread over the glass surface after perforation. Direct electrical contact either side of a single lipid bilayer membrane enables simpler interpretation of current–voltage relationships than when passing current across a closed GUV membrane. Consequently, Garten et al. were able to relate current characteristics directly to GUV membrane conductance as well as intrinsic system properties like selectivity (Garten et al. 2017). As the whole-GUV patch clamp configuration is analogous to basic planar bilayer configurations, we anticipate that many planar bilayer techniques could be adapted for whole-GUV patch clamp. However, currently only the most basic measurements (i.e. I-V curves) have been demonstrated using the whole-GUV method.

As electrical recording can be routinely made at microsecond resolution, whole-GUV patch clamping could be particularly suited for studying fast dynamics like pore opening and closing events, as have been observed using ion channels embedded in black lipid membrane (Winterhalter 2000). In contrast to planar bilayer measurements, patch clamping in GUVs also could excel in studying mechanosensitive ion channels as the membrane tension can be controlled by varying the aspiration pressure during electrical recording. However, this technique has not been widely adopted, likely due to its large technical overheads and limited throughput. Additionally, as several ions contribute simultaneously to recorded electrical currents, and measured circuit potentials differ from the transmembrane potential value, interpretation of patch clamping experiments using theoretical transport models might be challenging.

Monitoring specific ion concentrations with ion indicators—fluorescent molecules whose spectral properties are influenced by the presence of specific ions—offers an alternative to electrophysiological techniques (Valkenier et al. 2015; Tivony et al. 2022; Fletcher et al. 2022) (Fig. 2B). Decades of research into photochemistry have enabled the synthesis and discovery of a library of fluorescent readouts for physiologically relevant ions such as potassium, sodium, protons, and calcium (Meuwis 1995; Paredes 2008; Gao 2017; Sambath et al. 2021) as well as transition metals. Interested readers are referred to several excellent reviews focusing on ion indicators (Tsien 1989; Martínez-Zaguilán et al. 1996; Parvez et al. 2018; Li et al. 2022). Many of these fluorescent molecules can be routinely encapsulated inside GUVs during formation and included in the external solution (Valkenier et al. 2015; Tivony 2022;). Then, by using standard curves, ionic concentrations on both sides of the membrane can be directly determined from fluorescence measurements (Tivony et al. 2022; Ruppelt et al. 2024). As hundreds of GUVs can be visualised simultaneously under fluorescence microscopes, ion indicators allow highly parallel single vesicle studies of ion transport (Nahas et al. 2022; Fletcher et al. 2022). This has been exploited in several recent studies to measure distributions of transport parameters amongst GUV populations (Tivony et al. 2022).

A related method for studying ion transport dynamics is to use membrane voltage-sensitive fluorescent probes (Miller 2016). Various sensors exist including membrane soluble dyes (Garten et al. 2017), membrane associating DNA origami-based probes (Ochmann et al. 2021) and membrane protein probes (Bando et al. 2019; Ochmann et al. 2021), illustrated schematically in Fig. 2C. Additionally, probes which sense the local dipole field, such as di-8-ANEPPS and RH42, may also be used to monitor membrane voltage (Ramundo-Orlando et al. 2009). The molecular mechanisms of these are beyond the scope of this review but we refer interested readers to several excellent reviews (Miller 2016; Bando et al. 2019). Voltage-sensitive probes are very valuable in transport studies as they offer an approach for directly monitoring membrane potentials, which is vital for scrutiny of mechanisms in voltage-sensitive ion channels, for example. However, in order to disentangle the contribution of different ions to membrane potential buildup, it is essential to quantify the membrane permeability of each ion rather than measuring membrane potential alone.

 Unlike electrophysiological recording techniques which cannot distinguish between different ion species, fluorescence techniques can employ different ion indicators to probe the permeation of several ions at the same time. However, care must be taken to ensure ion indicators are suitable for studying a given transport process. For instance, fluorescence responses of chosen ion indicators can be slow and even rate limiting, prohibiting kinetic studies (Bando et al. 2019). Similarly, poor fluorescence responses may limit precise quantification of ion concentration with both widefield and confocal microscopes. Nevertheless, when appropriately used, ion indicators offer a direct method for quantifying the concentration of ions on both sides of the membrane (Ruppelt et al. 2024; Fletcher et al. 2022). In turn, accurate determination of both transmembrane ionic gradients and specific ion fluxes can be achieved (Tivony et al. 2022). In the following sections, we will discuss how the measurement of these variables enables precise estimation of transport parameters for different ion transport pathways.

## Analysing ion transport rates to elucidate transport mechanisms

Quantifying ion transport rates allows direct extraction of system parameters like permeability rates and transport selectivity. Intrinsic system parameters are important for two reasons. Firstly, they are dependent on specific membrane features (e.g., pore structure and lipid composition) and on the physicochemical nature of the permeated molecule. Consequently, these parameters can be measured to give insight into relationships between the structure of the transport system and its function (Roux 1996; Alcaraz et al. 2004). Secondly, once quantified, these parameters can be integrated into predictive theoretical models of ion transport pathway behaviour in new contexts, which is crucial for robust testing of biophysical theories (Alcaraz et al. 2004). Here, we will briefly review the most predominant models of ion transport processes applied to GUVs. Whilst more sophisticated models exist, these basic models constitute a guide for experimental design requirements for extraction of intrinsic parameters.

In the simplest model, for neutral species, transmembrane transport rates are typically understood as a special case of Fick’s first law, $J= -D\nabla C$, where $J$ is the flux density crossing the membrane, $D$ is the species’ diffusion constant across the membrane, and $\nabla C$ the transmembrane concentration gradient (Cama et al. 2015). Given the relevant ionic gradient is that across the membrane, which has a defined thickness, *L*, Fick’s law can be rewritten as follows: $J=P \Delta C$, where $P=\frac{KD}{L}$ and $\Delta C={C}_{\text{ext}}- {C}_{\text{int}}$ is the concentration difference across the membrane. As ions are charged species, their transport is also affected by electrostatic forces that arise from both membrane charge and charge separation across the membrane. A simple model that accounts for electrostatic forces due to transmembrane potential is the well-established Goldman–Hodgkin–Katz (GHK) model which relates *J *to concentration differences across the membrane as well as the transmembrane potential, V, through

1 $${J}_{i}= {P}_{i}\frac{VF}{RT}\frac{{{C}^{i}}_{int}-{{C}^{i}}_{ext}{e}^{-\frac{FV}{RT}}}{1-{e}^{-\frac{FV}{RT}}}$$

Where $i$ denotes the specific ion of interest, ${P}_{i}$ the permeability coefficient of ion $i$ through the membrane, and $F, R,$ and $T$ are the Faraday constant, molar gas constant, and temperature, respectively (Goldman 1943), with units Cmol^−1^, JK^−1^mol^−1^ and K respectively. ${{C}^{i}}_{\text{int}}$ and ${{C}^{i}}_{\text{ext}}$ represent the concentrations either side of the membrane. Limiting cases of the GHK flux equation have provided different recipes for quantifying membrane material properties like the permeabilities to different ions, ${P}_{i}$.

The first special case arises when the total current density across a membrane is zero, ${\sum }_{i}{J}_{i}=0$. This is the zero-current condition, or reversal potential condition. It can be measured by measuring I-V curves and finding the potential at which the measured current is zero (Alcaraz et al. 2004). This is most readily achieved using electrophysiology methods like patch clamping. The GHK equation at zero total current leads to the nontrivial solution

2 $$V= -\frac{RT}{F}\text{ln}\frac{{\sum }_{i}{P}_{i}{{C}^{i}}_{int}}{{\sum }_{i}{P}_{i}{{C}^{i}}_{ext}}$$.

Note this result reduces to the well-known Nernst equation when there is a single membrane permeable ion:

3 $$V=E-{E}_{0}= \frac{RT}{F}ln\frac{{\left[C\right]}_{int}}{{\left[C\right]}_{ext}}.$$

Using Whole-GUV patch clamping, for example, V, ${{C}^{i}}_{\text{int}}$ and ${{C}^{i}}_{\text{ext}}$ can be controlled, allowing extraction of ion selectivities, defined as ratios of permeabilities $\frac{{P}_{i}}{{P}_{j}}$. Garten et al. measured I-V relationships for membrane systems with and without gramicidin A, and with different concentrations of sodium and potassium on both sides of the GUV membrane (see Fig. 2D) (Garten et al. 2017). From the obtained I-V curves, the authors could directly relate the reversal potentials to the selectivity of each membrane system, highlighting how important mechanistic insight can be gained by quantitative measurements and modelling of isolated pathways.

The second limiting case is when the membrane potential is low $\left(V\ll \frac{RT}{F}\right)$. In this case, the GHK equation becomes equivalent to Fick’s first law:

4 $$J_i=P_i(C_{ext}^i-C_{int}^i)$$

where ${P}_{i }= \frac{KD}{L}$, where *K* is the partition coefficient, *D* is the diffusion constant for the ion within the membrane, and *L* the thickness of the membrane. This follows from applying L’Hopital’s rule to Eq. 1 in the limit $V \to 0$. This equation implies that, when V is low, the permeability rate of ion $i$ (${P}_{i}$) can be determined by measuring the flux ${J}_{i}$ at a defined concentration gradient value. Furthermore, for vesicle-based systems, it is possible to use continuity to relate the transmembrane flux ${J}_{i}$ to the rate of change of the internal concentration, as ${J}_{i}\times A=\frac{d{{C}^{i}}_{i\text{nt}}}{dt} ,$ where *A* is the vesicle surface area (assuming an isotropic flux). Using this relation, we can extract ${P}_{i}$ by solely monitoring the luminal (inside) and extravesicular (outside) concentration of ions. This framework for quantifying ${P}_{i}$ has led to several experimental strategies for monitoring both ${{C}^{i}}_{\text{int}}$ and${{C}^{i}}_{\text{ext}}$.(Deamer 1987; Tivony et al. 2022; Fletcher et al. 2022).

Extracting permeabilities from ionic gradients relies on the membrane potential being kept low $\left(V\ll \frac{RT}{F}\right)$. There are two main ways in which this can be achieved experimentally. One is to make the membrane very permeable to a ‘dummy ion’ which can transport across the membrane rapidly to compensate for V development caused by transport of an ion of interest (Dezi et al. 2013). For example, by using high background concentrations of potassium ions combined with highly specific potassium ionophores like valinomycin. Such an approach have been used in many studies to pin the membrane potential near zero to allow the extraction of proton permeabilities (Huang et al. 2021; Deamer 1987) and transport mechanisms (Lee et al. 2013). The second strategy is to estimate ${P}_{i}$ from early periods of transport processes. Typically, these experiments start with symmetric conditions across the membrane, meaning $V=0$. Ionic gradients can be generated by introducing the ion of interest on one side of the membrane. Transport of this ion and its counter ion at different rates results in charge separation and an associated increase in membrane potential. However, for some initial period, when $V\ll \frac{RT}{F}$, the flux obeys Fick’s first law and the permeability can be estimated. Tivony et al. demonstrated this approach for the first time by studying proton flux dependencies on concentration gradients (Tivony et al. 2022) (Fig. 2E). By carefully employing symmetric concentrations of the proton-indicator pyranine on either side of the membrane, accurate measurements of both $J$ and $\Delta [{H}^{+}]$ revealed a linear region during the early phase of transport. Moreover, Tivony et al. also demonstrated that estimating ${P}_{{H}^{+}}$ from early phase dynamics allows the membrane potential dynamics to be evaluated (Tivony et al. 2022). This strategy has the advantage over the use of ionophores as no additives are included in the membrane which may alter its properties. Notably, this approach relies on sub-millimolar ion detection sensitivities for extracting permeabilities from early phase transport (Fletcher et al. 2022). When detection sensitivity is limited to ion concentrations of > 1mM, the obtained flux profile (i.e., *J* vs. $\Delta$[ion]) can already be out of the linear regime before transported concentrations reach significant enough increases to elicit a fluorescent response.

## Reconstitution of different ion transport pathways in GUV systems

**Fig. 3:**
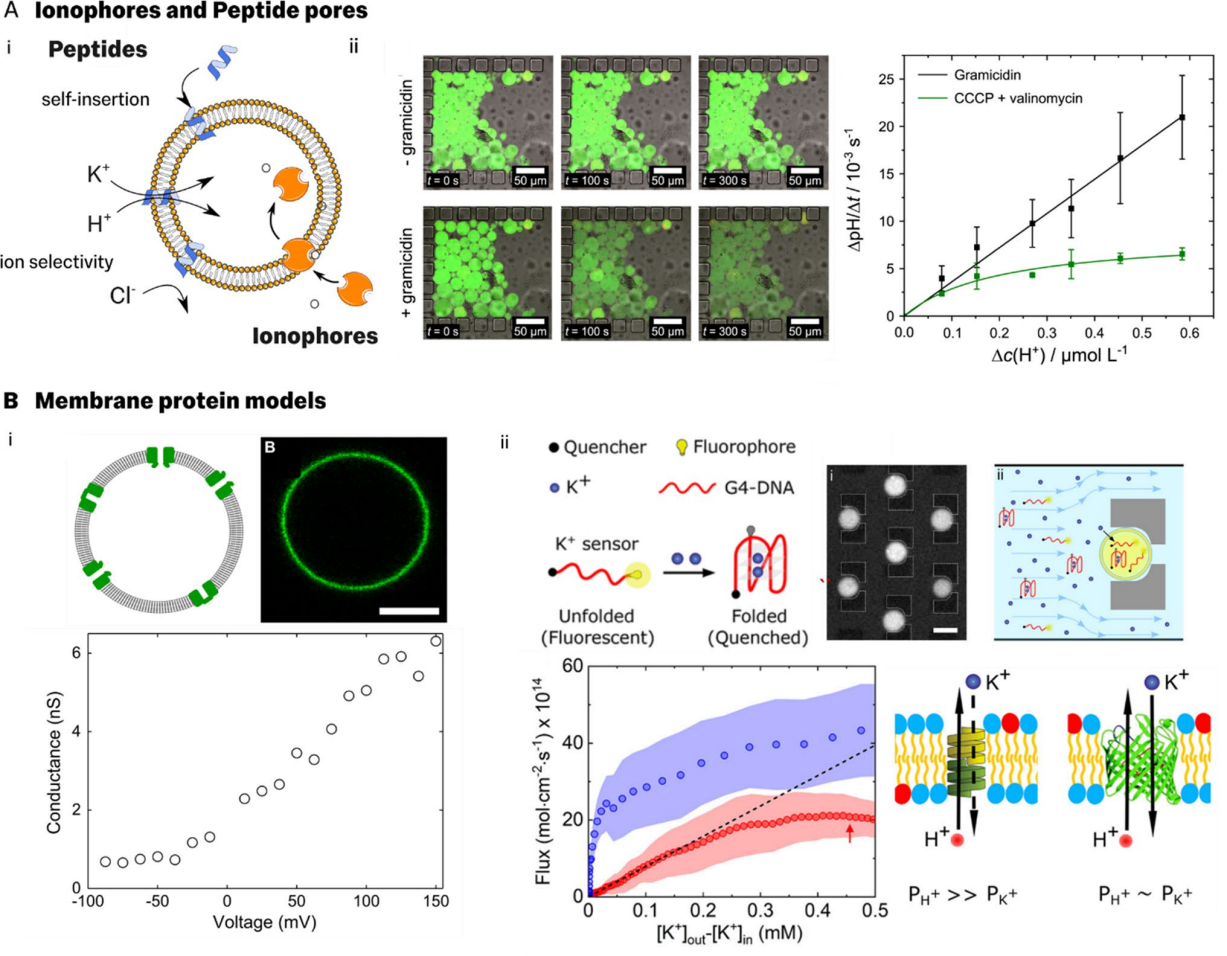
Experimental strategies for generating different facilitated ion transport pathways across GUV membranes** A**.**i** Ionophores (orange)—molecular carriers of ions across membranes like valinomycin and CCCP, and membrane active peptides (blue), such as Gramicidin A, which spontaneously embed in the lipid bilayer, are commonly used as model ion channels. These pores can display cell membrane protein reminiscent properties like ion selectivity(Finkelstein et al. 1981), Roux 1996). **ii**. Ruppelt et al. recently studied the proton transport characteristics of the pore forming ion channel gramicidin A (left)(Ruppelt et al. 2024). Then, the authors used their assay to establish archetypal proton transport characteristics for both pore forming peptides (gramicidin) and ionophores (valinomycin/CCCP) (right). Reproduced with permission from reference.(Ruppelt et al. 2024) **B** Membrane protein channels have also been used to study ion transport across GUVs. **i**. Aimon et al. reconstituted the voltage-sensitive ion channel KvAP (fluorescent labelled, shown in green, right) in GUV membranes. Using patch clamping of GUVs the authors determined the conductance for different applied voltages (bottom plot), indicating a gating behaviour. Reproduced with permission from reference (Aimon et al. 2011). **ii** Fletcher et al. reconstituted the outer membrane protein F from *E. coli* in GUVs and characterized its selectivity using an optofluidic potassium transport assay (Fletcher et al. 2022). The authors devised a novel potassium ion indicator using G-quadruplex forming DNA oligonucleotides, labelled with a fluorophore and quencher at each end of the DNA single strand. Presence of K + ions causes a transition in DNA structure from random coil to a folded G-quadruplex, resulting in a reduction in mean separation between fluorophore and quencher and an associated decrease in fluorophore fluorescence (left). Using microfluidic immobilisation of GUVs containing the DNA sensor (centre), the authors could develop K + ion gradients across GUV membranes and monitor the transmembrane transport of K + , for GUVs embedded with different model ion channels. The authors determined K + flux vs K + concentration difference relationships from K + transport dynamics for GUVs embedded with OmpF (blue) and gramicidin A (red). By analysing the non-linear characteristics of each profile using the GHK flux equation for K + influx and H + counter flux, the authors inferred a significantly lower selectivity (defined as) for OmpF than gramicidin A which reflects their differing pore sizes. Reproduced with permission from reference (Fletcher et al. 2022). Cartoons of lipids adapted from lipid-yellowgray icon by Servier https://smart.servier.com/ licensed without endorsement under CC-BY 3.0 Unported https://creativecommons.org/licenses/by/3.0/

Despite the promise of using GUVs to derive ion transport mechanisms, their broader application has so far been limited, mainly due to the difficulty of reconstituting membrane proteins like ion channels and pumps (Jørgensen et al. 2016). Instead, for over four decades, researchers have favoured simplified model ion transporters, such as the ionophores valinomycin and CCCP, to manipulate the concentration gradients of ions (e.g., potassium and proton, respectively) across lipid membranes (Fig. 3Ai) (Läuger et al. 1981; Kasianowicz et al. 1984; Kotova 2022). In particular, many ion transport studies using LUV suspensions have employed valinomycin and CCCP principally as a convenient tool to dissipate membrane potential gradients by allowing counter transport (Läuger et al. 1981; Deamer 1987; Paula et al. 1998). In contrast, very few GUV-based studies have directly studied the transport properties of ionophores. Notably, Valkenier et al. used GUVs to demonstrate the development of a novel chloride ionophore (Valkenier et al. 2015). It is likely, however, that many methods developed in LUV ionophore studies may be directly adapted for use in GUVs.

In addition to ionophores, commercial availability of short channel-forming peptides like gramicidin A has led to their wide use as model ion channels (Fig. 3Aii). Gramicidin A and other channel-forming peptides have been particularly favoured systems due to their water solubility and ability to efficiently self-insert in GUV membranes(Finkelstein et al. 1981). In contrast, purified membrane proteins require detergents to aid insertion. Despite their short amino acid sequence length, channel-forming peptides can be used to replicate important cellular ion transport phenomena, such as ion selectivity(Finkelstein et al. 1981; Deamer 1987; Fletcher et al. 2022). Additionally, due to their simplified peptide sequence, researchers have been able to probe relationships between molecular structure and transport behaviour (Fig. 3Aii) (Finkelstein et al. 1981; Fletcher et al. 2022). For example, measurements of ion transport across gramicidin A, reconstituted in liposomes, revealed its cation-selective nature and its high selectivity towards protons (Finkelstein et al. 1981; Ruppelt et al. 2024; Fletcher et al. 2022; Kelkar and Chattopadhyay 2007).

Some native protein ion channels and other water-filled channels have also been successfully studied in GUV systems. For example, Aimon et al. demonstrated the functional reconstitution of a voltage-gated potassium channel in GUV membranes (Aimon et al. 2011). In their study, the authors were able to observe gating by varying applied membrane voltages and measuring diode-like current characteristic using the patch clamp technique (Fig. 3Bi). Interestingly, the observation of gated currents implies a preferential orientation of the membrane protein, which occurs spontaneously (the authors acknowledge that they were not able to influence this). Additionally, Aimon et al. were able to measure single-channel opening and closing events in GUV patch clamp experiments, finding significantly more frequent opening events at positive applied voltages than negative voltages. Fletcher et al. studied transport of potassium across GUVs with reconstituted outer membrane protein F, (OmpF), a water filled channel with a pore size of ~ 1nm at its narrowest part (Fletcher et al. 2022). By comparing ${J}_{{K}^{+}}$ vs $\Delta [{K}^{+}]$ relationships for gramicidin A (pore size ~ 0.4 nm) embedded GUVs with OmpF equivalents, the authors were able to determine differences in specific cation selectivity between these two model systems and relate this insight to the known pore structures (Fig. 3Bii). These model ion transport pathways, and others successfully reconstituted into GUVs, are summarised in Table 2.

**Table 2:** Ion transporters reconstituted in GUV model membrane systems

Transporter	Transporter type	Biochemical class	Transport detection method	References
Gramicidin A	Ion channel	Peptide	Single-channel recording (SCR), fluorescence	( Goulian et al. 1998 ), ( Fletcher et al. 2022 ), ( Ruppelt et al. 2024 )
OmpF	Ion channel	Protein (*E. coli*)	Fluorescence, patch clamp	( Fletcher et al. 2022 ), ( Cama et al. 2015 )
KvAP	Voltage-gated ion channel	Protein (*Aeropyrum pernix)*	SCR	( Aimon et al. 2011 )
Bacteriorhodopsin	Light-stimulated proton pump	Protein (*Halobacterium salinarum*)	Fluorescence	( Dezi et al. 2013 )
AHA2	Proton pump	Protein (*Arabidopsis thaliana*)	Fluorescence	( Uzun et al. 2025 )
CCCP	Protonophore	Small molecule	Fluorescence	( Ruppelt et al. 2024 )
Valinomycin	K^+^ ionophore	Peptide	Fluorescence	( Ruppelt et al. 2024 )
SERCA	ATP-dependent Ca^2+^ pump	Protein (*rabbit muscle*)	Fluorescence	( Bian et al. 2016 )

A limitation of fluorescent GUV ion transport methods when applied to ion channels is that there are no general means to accurately determine the number of open channels in the membrane (Table 1) (Parvez et al. 2018). Furthermore, the time resolution of ion indicator responses may be longer than channel opening lifetimes (> ms), meaning discrete changes in transmembrane currents cannot be used to infer single channel events, in contrast to SCR (Fletcher et al. 2022). However, in principle, concentrations of reconstituted ion channels could be tuned such that only one ion channel exists on average per GUV, in which case permeability distributions over GUVs should have multiple peaks corresponding to *n* = 1,2,3,…. channels, which ought to follow Poisson statistics. From these distributions, the single-channel events could be isolated by identifying the population with the minimum peak in permeability. Then, as long as channel-mediated current remains the path of least resistance (as opposed to passive leakage through the lipid bilayer) single-channel permeabilities should be accurately quantified using present fluorescence-based GUV methods at reasonable timescales. For example, we may estimate the time taken for a single channel (conductance, ${G}_{{K}^{+}}\sim$ 20 pS at 0.1M KCl, 25 °C) to illicit a measurable concentration change in a GUV (radius ~ 10 µm) containing the DNA based K + sensor ( minimum observable change, Δ*C* ~ 50 µM) reported in Fletcher et al (2022). Based on known single channel conductance, we can estimate the single channel permeability to K + for gramicidin A as, ${P}_{{K}^{+}}\sim 1$ ms^−1^, using the relation:${P}_{{K}^{+}}=\frac{{G}_{{K}^{+}}RT}{{F}^{2}[{K}^{+}]{A}_{chan}}$, where F is the Faraday constant, [K^+^] = 0.1 M and ${A}_{\text{chan}}\sim 0.1$ nm^2^ (Fletcher, Zhu et al. 2022. Assuming a constant transmembrane K.^+^ gradient of ${\Delta [{K}^{+}]}_{\text{mem}}=1$ mM, we can calculate the rate of influx of K ^+^ using: $\frac{d[{K}^{+}]}{dt}=$ ${P}_{{K}^{+}}{\Delta \left[{K}^{+}\right]}_{\text{mem}}\frac{{A}_{\text{chan}}}{{V}_{G\text{UV}}}\sim 10 \text{nM}/\text{s}$ (or $36 \mu$ M/hr). This result implies that changes in internal GUV fluorescence (due to influx of ${K}^{+}$) can be detected after ~ 1 h, well within the experiment time used in Fletcher et al. (2022) to estimate the single-channel permeability of gA. Indeed, timescales on the order of hours are significantly longer than the measurement time required for accurate determination of single channel conductances using electrical measurements (seconds). However, this limitation is greatly compensated by the ability to measure 100s–1000s of GUVs in parallel using fluorescence microscopy (Fletcher et al. 2022).

Still, studying many aspects of cellular regulation, like voltage and chemical gating, requires specific proteins to be embedded in GUV membranes. However, limitations in the purification and reconstitution of membrane proteins prohibit their study. Whilst several reconstitution protocols exist, no one has emerged as a standard procedure. Detergent-mediated reconstitution and co-translational insertion using cell-free protein synthesis systems are promising options as they are compatible with a range of membrane proteins (Litschel and Schwille 2021; Harris et al. 2020). However, successful reconstitution using detergents can still critically depend on optimising a large number of parameters, such as detergent concentration, membrane composition, and electrolyte concentration (Croucht et al. 2018). Systematic optimisation of these methods is a slow process as GUV construction methods are laborious and typically restricted to a handful of compositions per reconstitution cycle. Increasing the throughput of reconstitution studies will be essential to allow the study of the numerous ion transport associated proteins in GUVs.

**Fig. 4:**
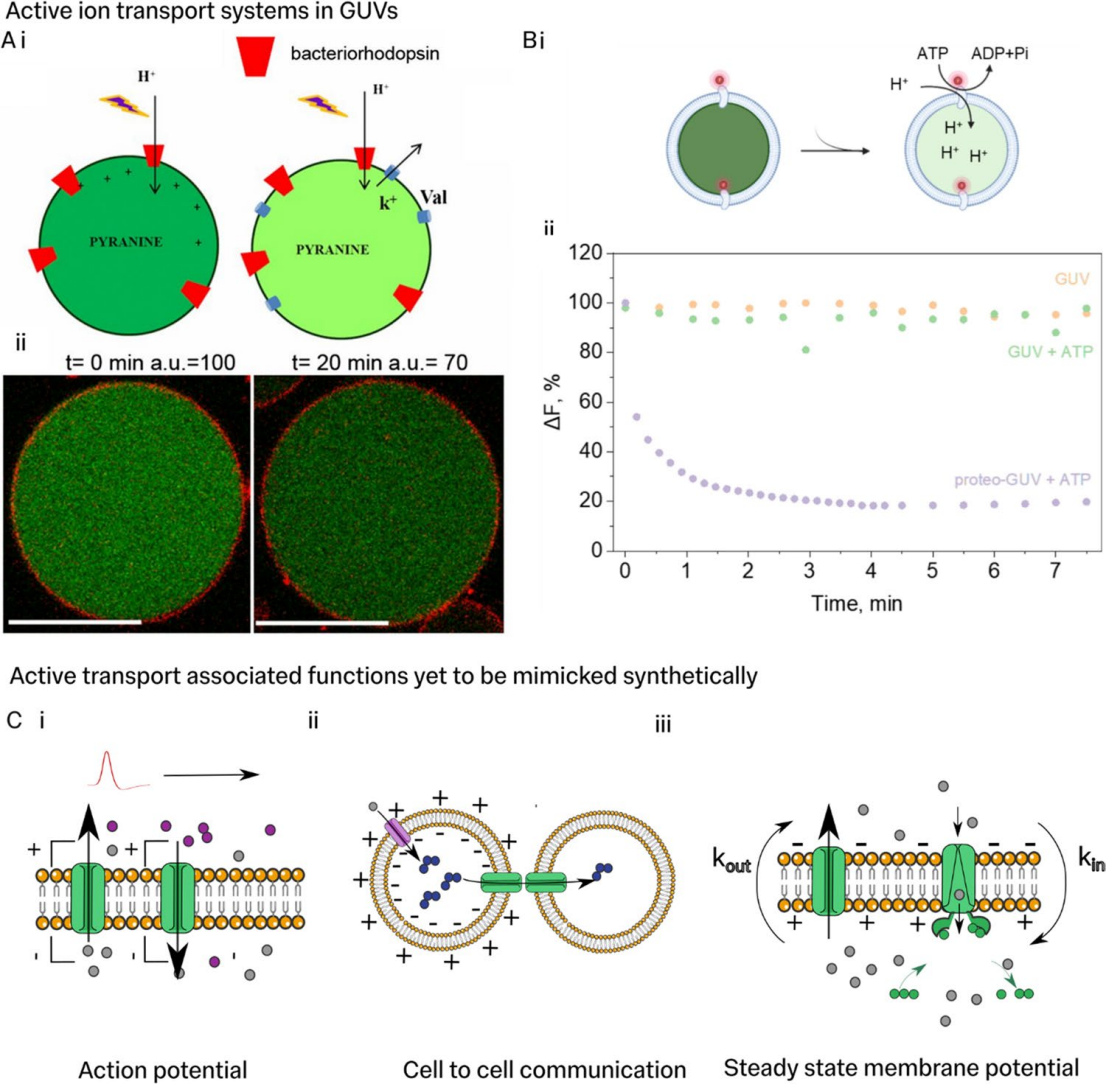
Coupling ion transport to function in artificial cells.** A** The photoresponsive proton pump, bacteriorhodopsin, was reconstituted into GUV membranes. **i** The fluorescent pH-sensitive dye, pyranine, was used to show accumulation of protons in GUV lumens due to proton pumping. Valinomycin was used to mediate membrane potential dissipation to maintain proton influx by bacteriorhodopsin. Note, proton transport into the GUV implies an excess of bacteriorhodopsins orientated such that proton pumping action transports protons inwards. **ii** Fluorescence intensity reduction of pyranine due to proton pumping activity of bacteriorhodopsin pores (Dezi et al. 2013).Reproduced with permission from (Dezi et al. 2013) (Copyright (2016) National Academy of Sciences. **B** Reconstitution and activity of *Arabidopsis thaliana* H^+^-ATPase isoform 2 (AHA2) in GUVs. **i** Schematic illustration of active transport of protons into pyranine loaded GUVs following activation of AHA2 by ATP. **ii** Pyranine fluorescence intensity of AHA2 reconstituted GUVs and GUVs following the addition of ATP. Fluorescence intensity reduction indicates that protons are actively pumped into the GUV lumen. Reproduced with permission from Uzun et al. (2025). Reproduced with permission from Fig. 4C in Uzun et al. under the CC licence (http://creativecommons.org/licenses/by/4.0/.) **C.** Illustrations of ion transport associated processes occurring in native cells which are yet, to our knowledge, to be demonstrated in artificial systems. **i** Action potential like travelling waves of membrane potential. **ii** Electrophoretic transport of charged molecules between cell compartments, used by cells as a form of signalling. **iii** Illustration of the steady state process of maintaining electrical potential by the coupling of passive and active transport processes. Cartoons of lipids adapted from lipid-yellowgray icon by Servier https://smart.servier.com/ licensed without endorsement under CC-BY 3.0 Unported https://creativecommons.org/licenses/by/3.0/

The GUV studies described thus far have all reconstituted passive transport pathways. Only very few examples of active ion transport studies in GUVs can be found in the literature. Light-responsive membrane proteins, which pump protons, such as proteorhodopsins, have proved a useful system for stimulating ion transport and have been included in GUV membranes (Dezi et al. 2013; Eaglesfield et al. 2021). Dezi et al. demonstrated that active proton transport could be initiated by the application of green light to GUVs with bacteriorhodopsin (also a proton pump) inserted into their membrane (Fig. 4A). However, in this study, only a proof of concept was demonstrated; the authors did not quantitatively study the active transport dynamics. Chemically driven ion pumping has also been achieved. A recent study demonstrated the ATP-driven pumping of protons across GUV membranes by reconstituting the plant-based proton pump AHA2 (Fig. 4Bi) (Uzun et al. 2025). The proton indicator pyranine was used to show transport of protons after the addition of ATP only when fluorescently labelled AHA2 was present in the membrane (Fig. 4Bii). So far, active transport studies have focused on proof-of-concept demonstrations. Integrating these new active transport systems into the quantitative assays described for passive transport should be straightforward. Achieving this will allow similar mechanistic insight into active transport as we have seen in passive transport pathways. Researchers have also begun to consider the molecular design of synthetic active ion transport pathways without leveraging proteins found in nature (Steinberg-Yfrach et al. 1997; Borsley 2024). Whilst still a nascent field, the realisation of completely synthetic active transport systems could bypass challenges of protein reconstitution.

## Outlook: towards functional ion transport elements in artificial cells

GUV-based ion transport assays have significant potential to be extended to gain much more insight into transport processes. One avenue to consider is to extend current fluorescence-based assays to include multiple fluorescent channels to monitor several important variables simultaneously. For example, membrane potential and ion concentrations could be monitored in parallel using well-separated spectral channels by employing recently published DNA-based sensors, whose fluorescent properties can be tuned to suit experimental requirements (Fletcher et al. 2022). There is also significant scope to develop electrical measurements of ion channels in GUVs. For example, whilst single-channel recordings under simultaneous membrane tension control have been demonstrated, only preliminary investigations into a limited number of channels have been reported (Goulian et al. 1998; Garten et al. 2017). Inspiration for further improving technical capabilities in electrical recordings of GUVs can be found in cell physiology trends, where microfluidics has been combined with multiplexed patch clamp to enable high-throughput single-cell measurements (Ionescu-Zanetti et al. 2005).

There is room for improvement in scaling up the complexity of GUV-based membranes as well. As more and more individual pathways are studied in isolation in GUVs, the combination of several transport systems will be able to model interactions between different pathways and elucidate emergent behaviour in coupled systems. In recent years, GUVs have become the state-of-the-art for artificial protein expression in cell-sized containers as purified gene expression systems can be readily included (Kuruma and Ueda 2015; Elani et al. 2015; Sampson et al. 2024). This is a potentially significant advantage of GUV systems over planar bilayers and LUVs as GUVs are uniquely able to combine reconstituted ion transport pathways and protein expression systems in the same cell model. A significant drawback in current artificial cell systems is the lack of ATP regeneration systems, which lead to short-lived expression (Berhanu et al. 2019). Studying the integration of proton gradient dependent ATP synthesis pathways could be essential for learning design rules for long-lifetime artificial cells. Studying ion transport systems in artificial cells could also enable classes of functions that have been entirely elusive in artificial systems, like long-range fast communication with action potential like electronic signals (Fig. 4Ci). Ion transport studies could also reveal how to engineer coupling of electrical sensing to cell-to-cell communication using electrophoretic transport across gap junctions (Fig. 4Cii) (Bolognesi et al. 2018). This understanding could lead to artificial cells which can sense living cell electronic state and respond, for example. Finally, coupled active and passive ion transport is also critical for the steady state of membrane potential and osmotic regulation that underlies living cell’s homeostasis (Fig. 4Ciii). Consequently, studying how to engineer ionic transport cycles will be critical for the development of sustained artificial cells.

## Conclusions

GUVs are emerging as a powerful tool for studying transmembrane ion transport mechanisms. Due to their micron scale, they naturally allow both single vesicle measurement and tight control of conditions either side of their membrane. In recent years, a suite of tools comprising both electrical patch clamping and fluorescent probes has been developed to allow precise monitoring of key transport variables like internal and external ionic concentrations and the membrane potential. Moreover, the development of GUV systems with model channel-forming peptides, ionophores, and membrane proteins has allowed the scrutiny of various aspects of cellular ion channel phenomena. Importantly, recent developments in quantitative transport studies have demonstrated how intrinsic transport parameters like permeability rate and selectivity can be accurately extracted. However, challenges in native membrane purification and reconstitution protocols still limit the types of ion transport mechanisms that can be studied in GUVs, particularly protein functions, such as voltage and chemical gating, and active systems like ion pumps. Resolving robust protocols for the reconstitution of these protein transporters will allow basic studies of how a protein’s function depends on basic biophysical variables. This, in turn, will pave the way for designing more sophisticated GUV-based systems that incorporate cell-like functionalities.

## Data Availability

No datasets were generated or analysed during the current study.
